# Relationship between CGM-derived nocturnal hypoglycemia and subjective sleep quality in people with type 1 diabetes

**DOI:** 10.1038/s41598-023-47351-x

**Published:** 2023-11-28

**Authors:** Daphne Gardner, Hong Chang Tan, Gek Hsiang Lim, May Zin Oo, Xiaohui Xin, Suresh Rama Chandran

**Affiliations:** 1 Department of Endocrinology, Academia, Singapore General Hospital, Level 3, 20 College Road, Singapore, 169 856 Singapore; 2 SingHealth-Duke NUS Diabetes Centre, Singapore General Hospital, Singapore, Singapore; 3 Health Sciences Research Unit, Singapore General Hospital, Singapore, Singapore; 4 Medicine Academic Clinical Program, Singapore General Hospital, Singapore, Singapore

**Keywords:** Diabetes complications, Type 1 diabetes

## Abstract

This pilot study explores the relationship between nocturnal hypoglycemia (NH) and subjective sleep quality in people with type 1 diabetes (T1D). Twenty-seven adults with T1D wore a Freestyle Libre Pro CGM and recorded subjective sleep quality daily, as assessed by a single Likert scale question. Frequency, duration, area under the curve (AUC) of NH (00:00–06:00) defined as sensor glucose below threshold (< 3.9 mmol/L; < 3 mmol/L) for ≥ 15 min, nocturnal mean glucose, Time in Range (3.9–10 mmol/L), and coefficient of variation were calculated. Twenty-seven adults, 18 (66.7%) women, with median (IQR) age of 27 (26, 32) years and HbA1c of 7.6 (7.1, 8.1) participated. Nights with NH < 3.9 mmol/L resulted in a lower (worse) sleep score than nights without NH [Mean (SD): 3.3 (1.2) vs 3.5 (1.0), p = 0.03). A higher frequency and longer duration but not AUC [adjusted OR (95% CI) 0.52 (0.38, 0.72), 0.961 (0.932, 0.991), 0.999 (0.998, 1.001) respectively)], of NH < 3.9 mmol/L, were associated with a lower sleep score. NH < 3.0 mmol/L metrics were not associated with sleep quality. Recurrent NH < 3.9 mmol/L, rather than prolonged NH < 3.0 mmol/L, seems associated with subjective sleep quality, implying that those with the highest burden of NH are likely unaware of it.

## Introduction

Sleep quality in people with type 1 diabetes is impacted by multiple factors. Chronic sleep disruption is highly prevalent among people with type 1 diabetes. A study in adults with type 1 diabetes showed that 17.7% wake up every night, and 53.5% wake up once or twice a week^1^. Nocturnal hypoglycemia (NH), nocturia due to hyperglycemia, and scheduled nighttime glucose checks could all lead to sleep disruption. Sleep disruption has multiple short-term and long-term consequences. Short-term consequences include being more irritable, distracted, and having less energy^1^. Short-term sleep disruptions also affect memory and learning^2^. Conversely longer-term consequences include a higher risk of glucose intolerance, hypertension, and a pro-inflammatory state, to name a few^3^. These are particularly important in people with type 1 diabetes at a higher risk of metabolic complications. Furthermore, as most people with type 1 diabetes are diagnosed young, sleep disruption could also impact their learning and work productivity.

Nocturnal hypoglycemia (NH) is a significant contributor to sleep disruption. Nocturnal awakenings due to symptomatic hypoglycemia result in obvious disruption to sleep and impact subjective sleep quality^4^. However, three-quarters of the NH are asymptomatic even below thresholds of 3 mmol/L and 2.2 mmol/L^5^. Some studies have shown that people with type 1 diabetes have a reduced threshold of awakening from NH and even increased sleep efficiency during NH^6,7^. A few other studies suggest that even asymptomatic hypoglycemia affects sleep quality^8–10^. While advances in diabetes technologies have reduced NH, they have had a variable impact on subjective sleep quality. Some studies using Real-time Continuous glucose monitoring (RT-CGM) and closed-loop technology have shown improved subjective sleep quality^11^. In contrast, others have not demonstrated any changes to subjective sleep quality^12,13^. Thus, overall, there is heterogeneity in the relationship of NH on sleep quality.

The heterogeneity of glycemic characteristics of nocturnal hypoglycemia may partly explain this. Continuous glucose monitoring has enabled detailed characterization of nocturnal hypoglycemia. However, most experimentally induced hypoglycemia has studied single episodes of prolonged hypoglycemia. Real-life nocturnal hypoglycemia is heterogeneous, with multiple short episodes or a few long episodes. Further hypoglycemia episodes could be of varying depths. It is unknown how these characteristics of nocturnal hypoglycemia affect sleep quality in people with type 1 diabetes. Little data exists on the impact of nocturnal hypoglycemia characterized by using a CGM on sleep quality. Available studies have not described nocturnal hypoglycemia characteristics in detail. To our knowledge, data on the impact of the duration, depth, and frequency of nocturnal hypoglycemia and nocturnal time-in-range and nocturnal glucose variability on sleep quality are lacking. Hence, we believe that these heterogenous characteristics of nocturnal hypoglycemia could varyingly impact the subjective sleep quality of a person with type 1 diabetes. We aimed to study the impact of these various attributes of nocturnal hypoglycemia on subjective sleep quality in people with type 1 diabetes.

## Methods

This was part of a pilot study whereby adults with type 1 diabetes were recruited from the Diabetes and Metabolism Centre, Singapore General Hospital. This study was approved by the SingHealth Central Institutional Review Board (Ref: 2021/2223). All study-related procedures were performed in accordance with the relevant guidelines and regulations. A prior publication from this cohort described the impact of smartphone-based activity tracking on nocturnal hypoglycemia^14^. We excluded those using continuous glucose monitoring and those with an estimated glomerular filtration rate < 30 mL/min/m^2^. Participants using an insulin pump without a CGM were eligible to participate. Participants wore Freestyle Libre Pro blinded CGM system and self-graded their sleep quality each morning based on "How well did you sleep last night?" using a 5-point Likert Scale (1—Very poorly, 5—Very Well). The responses were manually self-recorded by participants on questionnaires. Periodic reminders were sent to the participants by the study team, but there were no daily reminders. This question is modelled after a similar item in the Pittsburgh Sleep Quality Index^15^. This item has been used in prior studies and shown to have a good correlation with objective measures of sleep quality^16,17^. We chose a single question to limit responder fatigue from the need to respond to daily long questionnaires. We provided Abbott Freestyle Optium capillary glucose meters and test strips for participants to monitor capillary glucose as per their usual practice. Data from capillary glucose meters were downloaded at the end of the study. Participants also completed a questionnaire that collected demographic and diabetes-related information at recruitment. This included information about past severe hypoglycemia episodes and GOLD and DAFNE hypoglycemia scores. Gold score is a validated, 7-point Likert scale assessing the awareness of hypoglycemia. A higher score indicates reduced awareness of hypoglycemia. A Gold score of 4 or more is diagnostic of impaired hypoglycemia awareness^18^. DAFNE (Dose Adjustment for Normal Eating) hypoglycemia score is a 3-point descriptive score that assesses the threshold for symptomatic hypoglycemia^19^. A DAFNE Hypoglycemia score of 2 and 3 indicate detection of symptomatic hypoglycemia at a glucose level of < 3 mmol/L or never being able to detect hypoglycemia, respectively. A DAFNE hypoglycemia score of 2 and 3 indicates impaired awareness of hypoglycemia. Participants were instructed to avoid the use of Vitamin C supplements during study period as it is known to interere with the Freestyle Libre system^20^.

Nocturnal (0000–0600) hypoglycemia (< 3.9 mmol/L & < 3 mmol/L, for ≥ 15 min) episodes, duration, nocturnal mean glucose, Area under the curve (AUC), Time-in-Range (3.9–10 mmol/L), and glucose variability measured as coefficient of variation were calculated. AUC for hypoglycemia was calculated as the product of the absolute difference between hypoglycemic readings and hypoglycemia threshold multiplied by 15 min. Freestyle Libre Pro generates one sensor glucose reading every 15 min. The sum of these values for each night was the AUC. AUC incorporated both the duration and depth of NH. We excluded nights with less than 20 sensor readings (Nighttime 0000:0600, 24 sensor readings expected).

Data are presented as mean (standard deviation, SD), n (%), or median (interquartile range, IQR) based on normality of distribution. Comparison of nights with Level 1 only (< 3.9–3.0 mmol/L) and Level 2 (< 3 mmol/L) and Gold Scores across nights with and without nocturnal hypoglycemia was made using Independent Samples T Test or One-Way ANOVA (Kruskal Wallis) based on normality of distribution. Spearman's rank test was used to explore the correlation of Gold Scores with NH parameters. The chi-squared test was used for the comparison of proportions across groups. Relationships between sleep quality scores and glycemic indices were assessed using linear regression models. Given that each participant contributed daily data, such as sleep score and nocturnal hypoglycemia, there were anticipated to be many within-person fluctuations over time. Hence, mixed models were used, treating participants as a random effect. As sleep score was a 5-point Likert scale, a mixed-effects ordinal logistic regression was used to estimate the impact of nocturnal hypoglycemia (defined as whether it was present or absent, number of episodes, duration, AUC) on sleep score, treating participant as a random effect. Similarly, a mixed-effects ordinal logistic regression was used to examine the association of nocturnal mean glucose, nocturnal TIR, and sleep score. The models were adjusted for age and sex, as these are known to impact sleep scores^21^. We explored the relationship between nocturnal mean glucose and sleep score using the locally weighted running line smoother (LOWESS) function. All analyses were performed on Stata 17 (StataCorp. 2021. Stata Statistical Software: Release 17. College Station, TX, StataCorp LLC). A 2-sided p-value < 0.05 was used to declare statistical significance.

Additionally, we calculated the Mean Absolute Relative Difference (MARD) of the sensor glucose readings using the formula (|capillary glucose – sensor glucose|/capillary glucose) × 100. We used paired capillary and sensor glucose readings within 3 min of each other. Capillary glucose readings recorded manually after measuring on participants' capillary glucose meters were excluded as the accuracy of the testing time for these readings could not be verified. We used a total of 518 paired glucose readings, with glucose ranging from 1.1 to 27.8 mmol/L. However, only 58 paired glucose readings were in the glucose range of < 3.9 mmol/L. Clarke and Parkes error grid analysis was performed using the R package "ega". (https://cran.r-project.org/web/packages/ega/ega.pdf). We followed the recommendations by Clarke and Kovatchev^22^.

### Ethics approval

This study was approved by the SingHealth Central Institutional Review Board (Ref: 2021/2223).

### Consent

All participants provided an informed consent.

## Results

Twenty-seven adults with T1D, 18 (66.7%) women, participated. The cohort had a median age of 27 (IQR 26–32) years, mean BMI of 24 (3.4) kg/m^2^, median diabetes duration of 16 (IQR 12–20) years and median HbA1c of 7.6% (IQR 7.1–8.1). About half (14, 52.9%) used an insulin pump with Insulin Aspart. Among multiple daily insulin (MDI) users (13, 48.1%), the basal insulins used were U100 Glargine (6, 22.2%), U300 Glargine (6, 22.2%) and Detemir (1, 3.7%) and bolus insulin used were Aspart (7, 25.9%), and Glulisine (6, 22.2%). More than half (15, 55.5%) reported waking up at least once per week for NH before recruitment. The mean Gold score was 2.1, and 73.1% of participants could detect hypoglycemia ≥ 3 mmol/L. The median episodes of severe hypoglycemia in the past year and lifetime were zero. The baseline characteristics of the participants are in Table 1.

**Table 1:** Baseline participant characteristics.

Characteristic	Result
Age; Median (IQR)	27 (26, 32)
Age; Mean (SD)	30.6 (8.7)
Sex; n (%)	
Male	9 (33.3)
Female	18 (66.7)
Ethnicity; n (%)	
Chinese	22(80.8)
Malay	1 (3.9)
Indian	3 (11.5)
Others	1 (3.9)
BMI (kg/m^2^); Mean (SD)	24 (3.4)
Diabetes duration; Median (IQR)	16 (12, 20)
Diabetes duration; Mean (SD)	16.9 (7.3)
HbA1c, %; Median (IQR)	7.6 (7.1, 8.1)
HbA1c, %; Mean (SD)	7.7 (0.9)
HbA1c, mmol/mol; Median (IQR)	60 (54, 65)
HbA1c, mmol/mol; Mean (SD)	60.2 (9.5)
Treatment type; n (%)	
Multiple daily injections	13 (48.1%)
Insulin pumps	14 (51.9%)
Awaken due to hypoglycemia in the last 1 month; n (%)	
Never	12 (44.4)
1–2 times per week	12 (44.4)
3–4 times per week	3 (11.1)
Almost every night	0 (0.0)
Gold score; mean (SD)	2.1 (0.9)
Glucose level that hypoglycemia is recognized; n (%)	
≥ 3 mmol/L	19 (70.4)
< 3 mmol/L	8 (29.6)
Never	0 (0)
No. of episodes of severe hypoglycemia in lifetime; Median (IQR)	0 (0, 1)
No. of episodes of severe hypoglycemia in lifetime; Mean (SD)	1.3 (3.2)
No. of episodes of severe hypoglycemia in the previous year; median (IQR)	0 (0, 0)
No. of episodes of severe hypoglycemia in the previous year; Mean (SD)	0.2 (0.6)

In total, 54,831 sensor glucose readings (24 h) and 669 sleep scores were available. We used 573 pairs of nocturnal CGM data and next-day sleep scores for analysis, of which 137 (23.9%) nights had one or more NH episodes < 3.9 mmol/L. Of these, 89 (15.4%) had Level 1 NH (< 3.9–3.0 mmol/L) and 48 (8.4%) Level 2 NH (< 3 mmol/L). Each participant contributed between 8 and 37 nights for analysis. The median nights contributed by all participants was 28, and the mean (SD) of nights per participant was 26 (10.5). The total daily dose of insulin used by the participants was 43.9 (14) units per day and 0.66 (0.17) units/kg/day during the study period. There was no relationship between TDD and NH as reported previously^14^. The average Gold score of participants on nights with NH < 3.9 was marginally higher than on nights without NH < 3.9 [2.2(1.1) vs 2.0 (0.9), p = 0.016]. The Gold score had a weak positive correlation with the frequency of NH < 3.9 episodes (Spearman's rho = 0.11, p = 0.006). There was no significant correlation with duration or AUC of NH < 3.9 or NH < 3.0 parameters.

**Figure 1:**
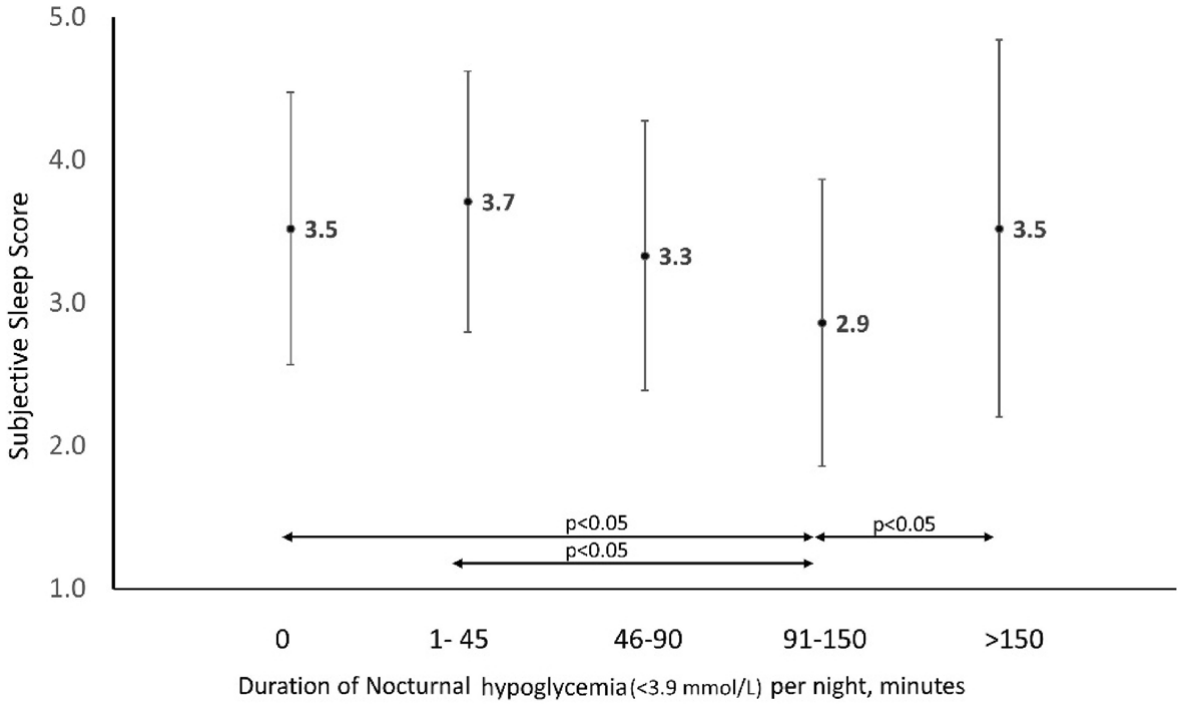
Comparison of Subjective sleep score across quartiles of nocturnal hypoglycemia (< 3.9 mmol/L) duration.

The mean sleep score following nights with NH was lower than on nights without NH [3.3 (1.2) vs 3.5 (1.0), p = 0.03]. A lower sleep score indicates worse subjective sleep quality. When sleep score was compared across nights without NH (0 min) and nights with NH divided into quartiles based on the duration of NH < 3.9 mmol/L (Quartiles I:1–45 min, II:46–90 min, III:91–150 min, IV: > 150 min), there was a progressive decline in sleep score across quartiles I, II and III. However, the mean sleep score for nights with the longest duration of NH < 3.9 mmol/L (> 150 min/night) was the same as for nights without NH. (Fig. 1). When nights with and without Level 2 NH (< 3 mmol/L) were compared, there was no significant difference between mean sleep scores [3.3 (1.2) vs 3.5 (1.0), p = 0.17].

A comparison of the characteristics of NH on nights with Level 1 vs Level 2 NH is shown in Table 2. The median duration of NH was 105 min for the whole cohort. Most of the nights analyzed had single episodes of hypoglycemia, while 11(8%) had two episodes every night. The total duration of NH was longer on nights with Level 2 NH compared to nights with only Level 1 NH [150 (105, 259) vs. 75 (45, 120) mins, p < 0.001]. The nocturnal mean glucose and the Nocturnal TIR were significantly lower (both p < 0.05), while the glucose variability measured as %CV was higher on nights with Level 2 NH (p = 0.08).

**Table 2:** Comparison of nights with Level 1 (3.8 – 3.0 mmol/L) vs Level 2 (< 3.0 mmol/L) Nocturnal hypoglycemia.

Characteristic	Overall (n = 137)	Nights with level 1 NH (3.8–3.0 mmol/L) (n = 89)	Nights with level 2 NH < 3.0 mmol/L (n = 48)	P value (nights with level 1 vs level 2 NH)
Duration of NH < 3.9 mmol/L in minutes/night;				
Median (IQR)	105(60,165)	75 (45, 120)	150 (105, 259)	< 0.001
Mean (SD)	128 (88)	98 (67)	183 (97)	< 0.001
Frequency of NH < 3.9 mmol/L; n(%)				0.22
1 episode/night	126 (92)	80 (89.9)	46 (95.8)	
2 episodes/night	11 (8)	9 (10.1)	2 (4.2)	
Area-under-curve of NH < 3.9 mmol/L (mmol/L.min);				
Median (IQR)	67 (27, 107)	36 (16, 69)	150 (85, 256)	< 0.001
Mean (SD)	99 (111)	49 (46)	193 (134)	< 0.001
Nocturnal mean glucose(mmol/L);				
Median (IQR)	5 (4.1, 6.3)	5.2 (4.6, 6.5)	4.3 (3.4, 5.7)	0.03
Mean (SD)	5.5 (2.1)	6.7 (1.8)	5.0 (2.4)	0.06
Nocturnal coefficient of variation (%)				
Median (IQR)	30.3 (22.1, 38.6)	27.4 (21.7, 37.4)	32.6 (26.6, 39.7)	0.082
Mean (SD)	31.2 (12.4)	30.1 (12.4)	33.2 (12.1)	0.16
Nocturnal time-in-range (3.9–10 mmol/L)				
Median (IQR)	62.5 (41.7, 75)	66.7 (52, 79.2)	41.7 (24.8, 62.9)	< 0.001
Mean (SD)	56.8 (23.8)	65.0 (19.3)	41.6 (24.1)	< 0.001

**Figure 2:**
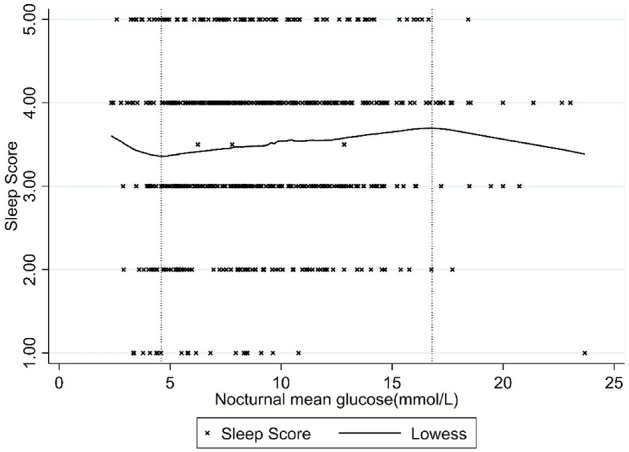
Relationship between nocturnal mean glucose and subjective sleep score. Inflection points indicated by dotted lines at 4.6 mmol/L and 16.8 mmol/L.

Mixed effect ordinal logistic regression showed that the presence of NH < 3.9 mmol/L was associated with a lower sleep score [OR 0.49 (95% CI 0.33, 0.74)] after adjusting for age and sex. A higher frequency of NH < 3.9 mmol/L episodes and total duration were associated with a lower sleep score [Adjusted OR 0.52 (95% CI 0.37, 0.72) and OR 0.961 (95% 0.932, 0.991), respectively]. AUC of NH < 3.9 mmol/L did not significantly impact sleep score. Similar parameters calculated for NH < 3.0 mmol/L did not impact sleep score. In addition, a higher nocturnal mean glucose was significantly associated with a higher sleep score [OR 1.06 (95% CI 1.01, 1.12), p = 0.01], while nocturnal %CV and Nocturnal TIR had no impact. (Table 3) A complex relationship was observed between nocturnal mean glucose and sleep score using the LOWESS smoothening. The sleep score increased with increasing nocturnal mean glucose but decreased after 16.8 mmol/L. At lower glucose levels, the sleep score paradoxically rose at low glucose levels when the mean nocturnal glucose was < 4.6 mmol/L (Fig. 2).

**Table 3:** Mixed-effect ordinal logistic regression of nocturnal glucose characteristics on sleep score, adjusted for age and sex.

Nocturnal glycemic characteristics	Adjusted OR (95% CI)	P-value
Nocturnal hypoglycemia (< 3.9 mmol/L)		
Presence	0.49 (0.33, 0.74)	0.001
Number of episodes	0.52 (0.37, 0.72)	< 0.001
Duration (per 15 min)	0.961 (0.9325, 0.991)	0.01
Area under curve	0.999 (0.998, 1.001)	0.43
Nocturnal hypoglycemia (< 3 mmol/L)		
Presence	0.67 (0.44, 1.02)	0.06
Number of episodes	0.71 (0.47, 1.08)	0.11
Duration (per 15 min)	1.00021 (0.962, 1.040)	0.99
Area under curve	1.001 (0.997, 1.005)	0.66
Nocturnal mean glucose	1.06 (1.01, 1.12)	0.01
Nocturnal %CV	0.99 (0.98, 1.001)	0.08
Nocturnal TIR	0.997 (0.992, 1.002)	0.20

The overall MARD for the sensor glucose readings compared to capillary glucose readings was 13.6%. The error grid analysis yielded 78.2% in Zone A, 19.7% in Zone B, and 0.2% in zone C. Overall, 98.1% were in the clinically acceptable zones on A-C.

## Discussion

We described the impact of characteristics of nocturnal hypoglycemia on subjective sleep score. We found that the presence, frequency, and duration but not depth of NH significantly impacted the subjective sleep quality in people with type 1 diabetes. However, nights with Level 2 NH, despite being much longer, were not associated with a lower sleep score.

Our cohort included 27 people with a median HbA1c of 7.4%, half using insulin pumps, all using insulin analogues receiving care in a tertiary center with access to a multidisciplinary care team and structured education. Despite this, 23.8% of the nights had NH with an impact on subjective sleep quality. In addition to the increased risk of diseases^3^ and consequent health care utilization, impaired sleep quality could have a ripple effect on the daily functioning of people with type 1 diabetes. A study in adults with T1D found that CGM-detected NH affects the mood the following day, resulting in less vigor, more fatigue, and more fluctuations in anger and vigor^23^. Impaired sleep quality has also been shown to impact learning ability and academic performance^24^. Further, it could also impact work productivity with absenteeism, personal job satisfaction, decision-making errors, team effectiveness, etc.^25^. Thus, our study highlights an unmet and possibly underrecognized need among people with type 1 diabetes to ensure optimal sleep quality and daytime functioning.

The heterogeneity of the impact of nocturnal hypoglycemia on sleep quality was evident from prior studies, with some showing that NH impacts sleep quality^6,7^, while others showing otherwise^8–10^. Our study reveals that at least part of this heterogeneity could be due to the heterogeneous nature of NH and its relationship to sleep quality as revealed by our study. Firstly, the frequency of NH < 3.9 mmol/L had a major impact, with nearly 50% odds of reduction of sleep score, while the impact of the duration of NH was much lower. Previous studies have reported similar findings on the impact of NH on subjective sleep scores^26^. However, most experimental studies mimic prolonged NH, lowering and holding the glucose level in the hypoglycemia range for a prolonged duration, but not recurrent episodes of NH. Secondly we calculated AUC, which incorporates both the depth and duration of NH. However, AUC did not significantly affect sleep score, suggesting a lack of impact of depth of hypoglycemia on sleep score. Findings in children with overnight polysomnography support this. Glucose measurements every 15 min found that even profound hypoglycemia with a median glucose nadir of 2 (IQR 1.4–3.3) mmol/L did not affect sleep architecture or next-day cognitive function^27^. Similarly, in our study, glycemic parameters of Level 2 NH (< 3 mmol/L) did not impact sleep score. This is despite the much longer durations of NH on nights with Level 2 NH. More than half of the nights with Level 2 NH had prolonged hypoglycemia of > 120 min. Thirdly, we found a complex relationship between nocturnal mean glucose and sleep score. The sleep score decreased (got worse) when nocturnal mean glucose was above 16.8. Hyperglycemia-mediated glucosuria and nocturia leading to sleep disruption could explain this. However, a paradoxical increase (better) sleep score was seen with a nocturnal mean glucose < 4.6 mmol/L. If confirmed in future studies, this is an interesting finding and may suggest a multiphasic relationship between nocturnal mean glucose and subjective sleep quality. A potential explanation is the deepening of sleep documented in children with type 1 diabetes during hypoglycemia^7^. Other studies have also suggested a suppression of counter-regulatory responses during sleep in people with T1D, related to impaired awareness during wakefulness^6^. We could not test this as all participants in our study had a Gold score of 4 or lower, indicating good awareness of hypoglycemia.

Thus in summary, our findings suggest that recurrent episodes of Level 1 NH have a higher impact on subjective sleep quality than prolonged episodes of Level 2 NH. Furthermore, nocturnal mean glucose may have a multiphasic relationship with sleep quality, with a possible paradoxical increase in sleep quality at very low glucose levels. Future studies exploring the relationship between NH and sleep should characterise NH in greater detail, describing NH's frequency, duration, levels and AUC.

Asymptomatic NH is well documented with the increasing use of CGM. We used a blinded or professional CGM as our focus was to study the natural behaviour of NH among people using multiple daily insulin injections or insulin pumps without RT-CGM. Hence, we excluded people using RT-CGM from this study. Although the use of RT-CGM is increasing, more than half of people with T1D worldwide do not use RT-CGM^28^. Data suggest that up to three-quarters of CGM-detected hypoglycemia is asymptomatic; hence, most people may sleep through NH episodes^5^. In our study, nights with Level 2 NH had a median duration of 150 min, and 95.8% of them were single episodes. Despite this, Level 2 NH had no impact on subjective sleep scores. This suggests that people with type 1 diabetes may sleep through deep and prolonged episodes of hypoglycemia without any subjective awareness of the impact on sleep quality the following day. Our findings align with recent reports suggesting that only symptomatic nocturnal hypoglycemia affects sleep quality^29^. It is plausible that nights with recurrent Level 1 NH impact awareness and possible awakening, while prolonged Level 2 NH suggests nights where the person slept through NH. Indeed, we found that the mean sleep score for nights in the highest quartile of NH duration (> 150 min) was not different from nights with no NH. Further, a survey of adults about self-detected nocturnal hypoglycemia found a high negative impact on quality of life the next day^26^. This lack of subjective awareness of deep and prolonged NH is critical.

Deep and prolonged NH have various health consequences for the person with type 1 diabetes. Prolonged NH is associated with the lengthening of QT intervals and bradycardia^30^. It is well recognized that such prolonged episodes can increase the risk of fatal arrhythmia, especially in a person with underlying high cardiovascular risk^31^. Further, prolonged NH increases the risk of impaired awareness of hypoglycemia (IAH)^32^. Development of IAH initiates a vicious cycle that predisposes the person to have further episodes of daytime and NH, further worsening the IAH. Thus, detecting and preventing prolonged Level 2 NH is essential from a clinical perspective, although they seem to have little impact on subjective sleep quality. However, our data suggests that such episodes may not be self-evident to the person with type 1 diabetes as they may not notice any subjective change in sleep quality. Hence, even a carefully taken history cannot detect the very cohort at the highest risk of NH among those with type 1 diabetes. This reiterates the need for continuous glucose monitoring in people with type 1 diabetes to detect and prevent NH.

The strengths of our study include the relatively large number of nights with paired sleep scores available for analysis. We evaluated nocturnal hypoglycemia in more detail than described before. The significant limitations of our study include the lack of data on nocturnal awakenings or symptoms and, hence, our inability to differentiate whether the NH was symptomatic. We only used a single-item questionnaire to evaluate sleep quality to reduce the questionnaire burden. We acknowledge the lack of detailed characterization of sleep quality. We did not adjust for other potential confounders of sleep quality like alcohol intake, evening exercise, pre-existing insomnia or shiftwork. However, participants reported taking > 2 units of alcohol only on 17/573 nights. Findings from our study may not apply to people using real-time CGM with alarms as the alarms may modify the relationship we found. Further, this analysis did not account for the impact of weekdays vs. weekends. Finally, this was a pilot study with only a few participants, and hence the findings should be viewed as exploratory and need confirmation in a larger sample.

## Conclusion

We found that recurrent episodes of Level 1 nocturnal hypoglycemia, rather than prolonged episodes of Level 2 nocturnal hypoglycemia, impact subjective sleep quality in people with type 1 diabetes. This implies that people with the highest burden of NH are likely to be less aware of it and, hence, challenging to diagnose based on symptoms. We also present the complex relationship between characteristics of nocturnal hypoglycaemia and subjective sleep quality, thereby highlighting the need for more detailed characterisation of NH in future studies.

## Data Availability

The datasets generated during and/or analyzed during the current study are not publicly available due to Institutional data protection policies, but are available from the corresponding author on reasonable request.
